# Anti‐inflammatory mechanisms and research progress of colchicine in atherosclerotic therapy

**DOI:** 10.1111/jcmm.16798

**Published:** 2021-07-27

**Authors:** Yuyu Li, Yuxin Zhang, Jianrong Lu, Yong Yin, Jun Xie, Biao Xu

**Affiliations:** ^1^ Department of Cardiology Nanjing Drum Tower Hospital MOE Key Laboratory of Model Animal for Disease Study School of Medicine Nanjing University Nanjing China; ^2^ Institution of Translational Medicine Drum Tower Hospital Medical School of Nanjing University Nanjing China

**Keywords:** atherosclerosis, colchicine, NLRP3 inflammasome

## Abstract

Inflammatory responses play a vital role in the onset and development of atherosclerosis, and throughout the entire process of the chronic disease. The inflammatory responses in atherosclerosis are mainly mediated by the NLRP3 inflammasome and its downstream inflammatory factors. As a powerful anti‐inflammatory medicine, colchicine has a history of more than 200 years in clinical application and is the first‐choice treatment for immune diseases such as gout and familial Mediterranean fever. In atherosclerosis, colchicine can inhibit the assembly and activation of NLRP3 inflammasome via various mechanisms to effectively reduce the expression of inflammatory factors, thereby reducing the inflammation. Recent clinical trials show that a low dose of colchicine (0.5 mg per day) has a certain protective effect in stable angina patients or those with acute myocardial infarction after PCI. This article summarizes and discusses the mechanisms of colchicine in the treatment of atherosclerosis and the latest research progress.

## 1 INTRODUCTION

The onset and progress of atherosclerosis are closely related to aseptic inflammation. The inflammatory responses in atherosclerosis are mainly developed through the NLRP3 inflammasome, interleukin‐1 beta (IL‐1β) and interleukin‐6 (IL‐6) inflammatory response axis and eventually lead to an increase in the C‐reactive protein (CRP). Colchicine can block NLPR3 inflammasomes through a variety of ways, thereby inhibiting downstream pathways and reducing the inflammatory responses in atherosclerosis.^1^, ^2^, ^3^ Although lipid‐lowering therapy is still the cornerstone, anti‐inflammatory therapy is opening up new ways to treat atherosclerosis. According to a large number of clinical studies in recent years, colchicine, as an anti‐inflammatory drug, is increasingly present in cardiovascular disease treatment programmes.

## 2 INFLAMMATORY RESPONSES IN ATHEROSCLEROSIS

In the early 19th century, pathologists Rokitansky and Virchow put forward the view that atherosclerosis is closely related to inflammation.^4^ However, this view did not attract enough attention at the beginning of the 20th century. According to the traditional view, coronary atherosclerosis is caused by the continuous deposition of lipids under the endothelium of blood vesselsalone.^5^ Until the 1990s, as atherosclerosis research progressed, the role of inflammation in the progression of coronary atherosclerosis became more prominent.

### 2.1 Lipoproteins deposited subcutaneously in blood vessels activate inflammatory responses

There are many hypotheses about the mechanism of atherosclerosis, and one widely accepted among them is the endothelial injury theory. The hypothesis suggests that a disorder haemodynamics or hypoxia‐affected local vasculature could lead to vascular endothelial damage, and apolipoproteins carrying cholesterol can continuously deposit under vascular endothelium with blood circulation.^6^ The lipoproteins are very easily oxidized under the intima of blood vessels and mainly composed of oxidized low‐density lipoprotein (OxLDL) and cholesterol crystals.^7^ OxLDL can induce leukocyte recruitment and activation to promote inflammation. OxLDL could also activate macrophages through the CD36‐TLR4‐TLR6 complex to promote NLRP3 inflammasome‐related inflammatory responses.^8^ By activating the NLRP3 inflammasome in ApoE^−/−^ mice, the increase in cholesterol crystals was positively correlated with the increase in macrophages. Furthermore, in the experiments on NLRP3^−/−^, ASC^−/−^, IL‐1α^−/−^and IL‐1β^−/−^ transgenic mice, the author found that atherosclerotic plaques were significantly reduced, inflammation levels alleviated. Those could confirm that the atherosclerotic inflammatory response induced by cholesterol crystals was closely related to the activation of the NLRP3 inflammasome and the level of interleukin‐18 (IL‐18) and IL‐1β could significantly decrease with the formation and activation of the NLRP3 inflammasome.^9^ In general, when the vascular endothelium is damaged, the deposition of lipoproteins under the vascular endothelium could induce inflammation subsequently in atherosclerotic plaques by inducing the NLRP3 inflammasome.

### 2.2 NLRP3 inflammasome, IL‐1 β, IL‐6, C‐reactive protein inflammatory response axis

In 2002, Fabio Martinon et al. first identified a caspase‐activating complex and named it the inflammasome.^10^ From then on, many kinds of inflammasome have been observed and reported. Different pathogen‐associated molecular patterns (PAMP) and damage‐associated molecular patterns (DAMP) can induce inflammatory response by activating different inflammasomes.^11^ the NLRP3 inflammasome is a very important member of the inflammasome family and plays a crucial role in atherosclerotic inflammation.

#### 2.2.1 Expression of NLRP3 inflammasome

**FIGURE 1:**
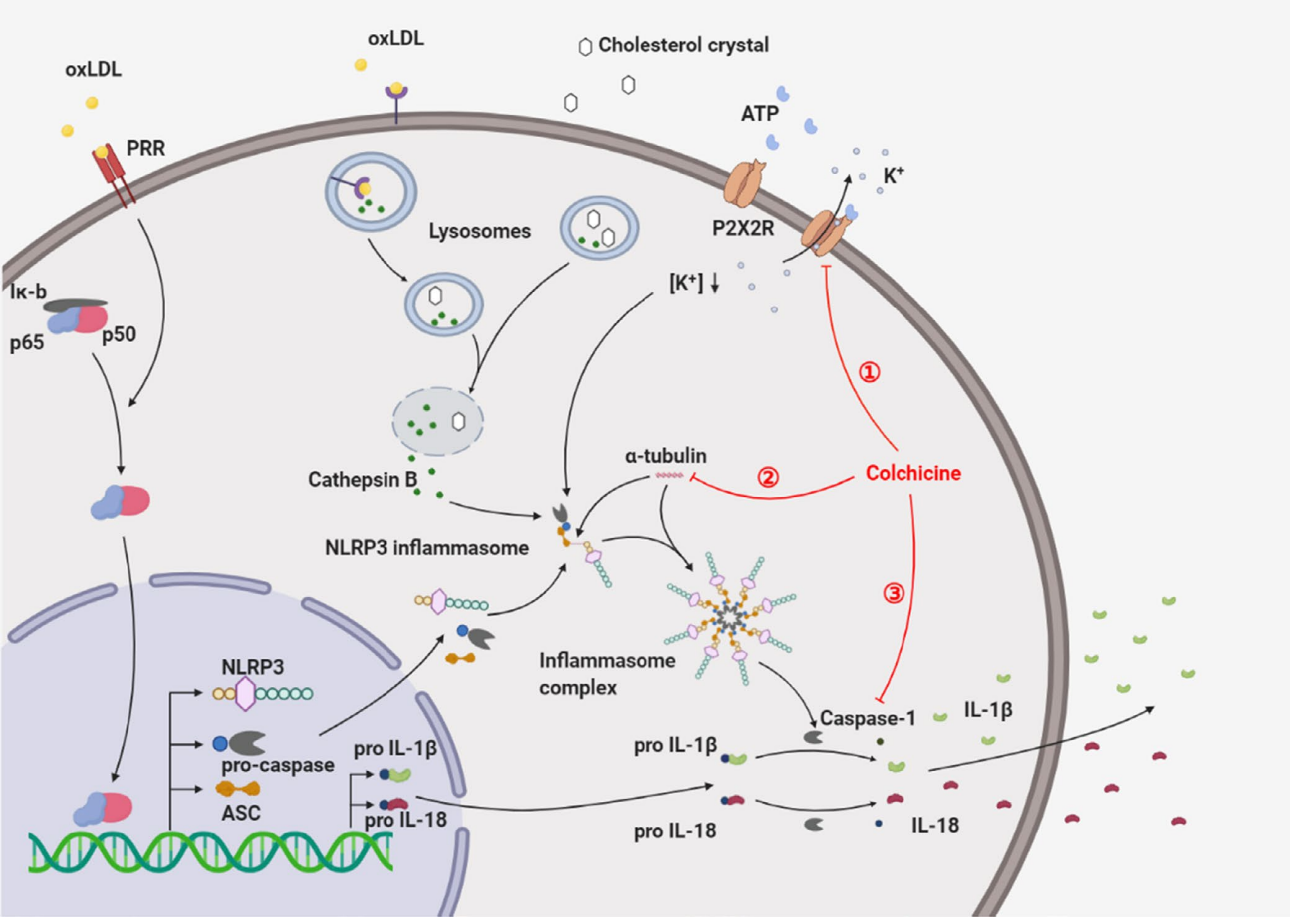
Inflammasome assembly and activation mechanism. OxLDL can activate the NF‐κB signal transduction pathway through PRR, which not only increases the expression of NLRP3, pro‐Caspase and ASC, but also upregulates the pro‐IL‐1β and pro‐IL‐18 levels. Extracellular ATP can combine with P2X2R to activate P2X2R, increase K^+^ efflux and decrease intracellular K^+^ concentration, which provides a basis for the assembly and activation of NLRP3 inflammasomes. At the same time, OxLDL can be swallowed by macrophages through membrane receptors and can be converted into cholesterol crystals in the lysosome. The CCs (formed intracellularly or derived extracellularly) can cause the lysosome to rupture, resulting in Cathepsin B being released into the cytoplasm and inflammasome activation. Microtubules can transport ASC so that it can combine with NLRP3 and assemble NLRP3 inflammasomes into complexes. After the inflammasome is activated, pro‐Caspase can be converted into Caspase by shearing, and the activated Caspase can cleave pro‐IL‐1β and pro‐IL‐18 into IL‐1β and IL‐18, and secrete them outside the cell, causing an outbreak of inflammation. However, colchicine can inhibit the activation of NLRP3 inflammasomes and reduce the release of IL‐1β through a variety of ways to inhibit inflammation, mainly in three ways as follows: ① restriction of P2X7 receptor and reduction of K^+^ outflow; ② damping of microtubule synthesis, and inhibition of the assembly of NLRP3 inflammasome and NLRP3 inflammasome complex; ③ inhibition of NLRP3 inflammasome activation and IL‐1 β release

In general, NLRP3 inflammasomes are expressed in myeloid cells, such as monocytes, neutrophils and eosinophils.^12^ The expression of NLRP3 inflammasomes is activated by PAMP and DAMP. Rosenfeld ME and Campbell LA reported that infections of different pathogens, including bacteria and viruses, can promote and aggravate the inflammatory response by activating the NLRP3 inflammasome, thereby increasing the risk of cardiovascular disease.^13^ Both PAMP and DAMP activate the downstream NF‐κB signal transduction pathway through the pattern recognition receptor (PRR) to promote the NLRP3 inflammasome expression. In atherosclerosis, OxLDL can directly activate the downstream NF‐κB signal transduction pathway through DAMP to increase the NLRP3 inflammasome of the expression, ASC (apoptosis‐associated speck‐like protein containing a CARD), pro‐IL‐1 β and pro‐IL‐18 (Figure 1).

#### 2.2.2 Assembly and activation of NLRP3 inflammasome

At present, it is believed that the activation of the NLRP3 inflammasome is extremely dependent on the intracellular K^+^ concentration: only when it is less than 70 mM can the NLRP3 inflammasome be assembled and activated. When P2X7 receptors are activated by extracellular ATP, they open K^+^ channels and a massive moment of K^+^ flows out. The decrease in intracellular K^+^ concentration provides a basic prerequisite for the assembly and activation of the NLRP3 inflammasome.^14^, ^15^ The proteins induced by NF‐κB signal transduction pathway, such as NLRP2 and ASC, could then assemble with pro‐caspase in the cytoplasm to form the NLRP3 inflammasome.^16^ Among them, the micro‐tubule plays a critical role in the assembly of NLRP3 and ASC.^17^ In addition, cholesterol crystals (CCs) deposited under the damaged endothelium can be swallowed into lysosomes in macrophages through endocytosis, while OxLDL can also be swallowed by macrophages through receptor‐mediated endocytosis. OxLDL can be converted into cholesterol crystals in the lysosome to form intracellular CCs. Either intracellular or extracellular CCs can result in lysosomal membrane instability and rupture.^9^ At this time, a large amount of cathepsin B in lysosomes will be released into the cytoplasm, thus inducing the activation of the NLRP3 inflammasome.^16^, ^18^ Other common upstream NLRP3 inflammasome activation mechanisms include mitochondrial damage, release of cardiolipin and mitochondrial DNA, and release of reactive oxygen species^19^, ^20^, ^21^ (Figure 1).

#### 2.2.3 Activation of IL‐1 β, IL‐6 and C‐reactive protein

**FIGURE 2:**
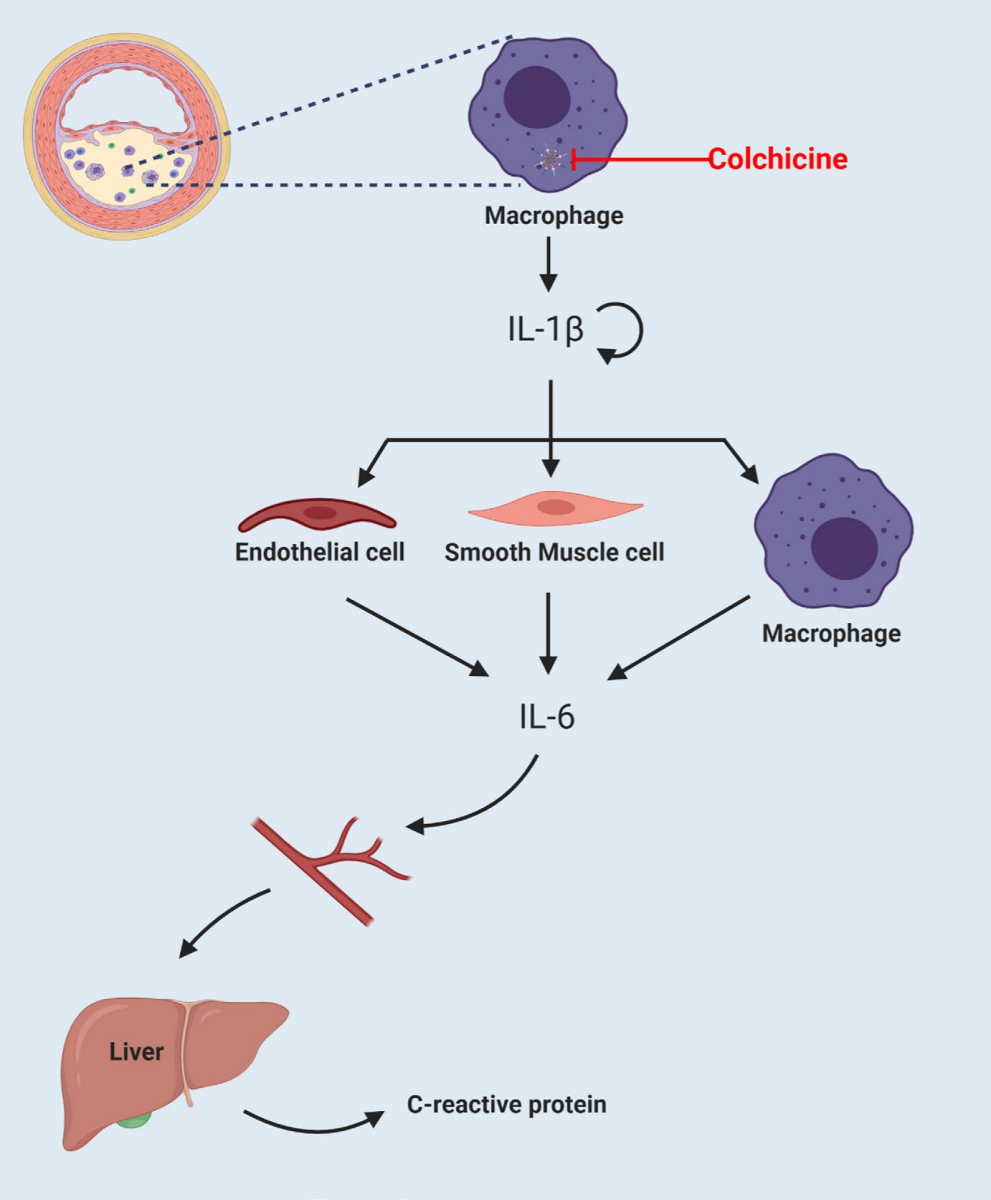
NLRP3 inflammasome, IL‐1 β, IL‐6, C‐reactive protein inflammatory response axis. The activation of NLRP3 inflammasomes in macrophages can activate caspase‐1 and release a large amount of IL‐1β, which can induce inflammatory factor storms through self‐activation. In addition, IL‐1β can activate endothelial cells, smooth muscle cells and macrophages to release a number of IL‐6. IL‐6 can circulate through the blood to the liver and induce hepatocytes to produce CRP. The marker of CRP as an indicator of clinical inflammation can be detected in patient blood samples. With CRP ≥2 mg/L, we can consider there to be an inflammatory response in the patient's body. Colchicine can inhibit the activation of NLRP3 inflammasomes, thereby causing the downstream levels of IL‐1β, IL‐6 and CRP to decrease

NLRP3 inflammasome activation will induce the splicing of pro‐caspase itself and activate caspase 1. Activated caspase 1 can cleave pro‐IL‐1β and pro‐IL‐18 to form mature forms of IL‐1 β and IL‐18, which can be secreted out of the cell.^22^ Extracellular IL‐1β and IL‐18 can trigger a cascade of inflammatory factors and amplify inflammation through self‐activation.^23^, ^24^ IL‐1β and IL‐18 are relatively upstream cytokines in the inflammatory pathway. They can stimulate the secretion of other cytokines, increase the recruitment of leukocytes and promote inflammation.^25^ On the other hand, IL‐1β and IL‐18 can stimulate a variety of cells such as macrophages, vascular smooth muscle cells and endothelial cells to produce large amounts of IL‐6.^23^, ^24^ Daniel J. T yrrell et al. believed that IL‐6 can accelerate the formation of atherosclerosis by aggravating mitochondrial dysfunction in vascular smooth muscle cells.^26^ A large amount of IL‐6 can enter the liver through blood circulation and stimulate hepatocytes to synthesize acute‐phase reactants, such as fibrinogen and plasminogen activator inhibitor. It can also induce the liver to synthesize a marker of inflammatory state: C‐reactive protein.^27^, ^28^ As early as 1997, Paul M. et al. used the detection of plasma C‐reactive protein levels to predict the risk of patients’ future myocardial infarction and stroke.^29^ It is currently believed that hsCRP level of greater than or equal to 2 mg/L can be considered as an inflammatory response (Figure 2).

## 3 ANTI‐INFLAMMATORY MECHANISMS OF COLCHICINE IN ATHEROSCLEROSIS

Colchicine, as a powerful anti‐inflammatory drug, has been used for rheumatic immune diseases for many years, as well as gout, familial Mediterranean fever and osteoarthritis. Compared with non‐steroidal anti‐inflammatory drugs and antibiotics, colchicine has a different anti‐inflammatory effect.

### 3.1 Inhibition of neutrophil chemotaxis, adhesion and recruitment

In the early stage of inflammation, neutrophils, as the forerunner of immune responses, can be affected by chemokines and then reach the inflammatory site first through blood circulation, and adhere to vascular endothelial cells though E‐selectin and L‐selectin. Through deformation, neutrophils can go through the intercellular space to reach the site of inflammation. Phelps P et al. discovered that in vitro, 0.1 nM colchicine can inhibit the chemotaxis of neutrophils and inhibit the release of chemokine S100A8 and S100A9 in neutrophils.^30^ What's more, Cronstein B et al. found that colchicine can also inhibit neutrophil adhesion and recruitment by inhibiting microtubule synthesis and promoting microtubule depolymerization.^31^, ^32^ Colchicine at 300 nM can directly cause the exfoliation of the adhesion molecule L‐selectin on the surface of endothelial cells and hinder the recruitment of neutrophils.^32^ Colchicine can also inhibit the synthesis of superoxide in neutrophils by inhibiting microtubules, thus reducing inflammation.^2^, ^33^ In vitro 3 nM colchicine can change the distribution of E‐selectin on the surface of endothelial cells and inhibit the adhesion of neutrophils. It is because colchicine can inhibit the chemotaxis, adhesion and recruitment of neutrophils that its clinical dosage needs to be precise. A high dose will often cause myelosuppression and neutropenia, therefore resulting in infection.

### 3.2 Inhibition of NLRP3 inflammasome activation and IL‐1 β release

Colchicine can inhibit not only neutrophils, but also NLRP3 inflammasome activation in many ways, thus exert a powerful anti‐inflammatory effect. Martinon F. et al. proved that colchicine can inhibit the activation of the NLRP3 inflammasome in cultured monocytes.^34^ Current studies have shown that the mechanism of colchicine on the NLRP3 inflammasome can be summarized into the following three types.

#### 3.2.1 Colchicine can restrict P2X7 receptor

Marques‐da‐Silva C et al. evaluated the function of heterologous P2X2 and P2X7 receptors after ATP treatment by electrophysiology and dye uptake, and verified it with colchicine in vitro. The results showed that colchicine could effectively inhibit the pore formation induced by P2X7.^35^, ^36^ As mentioned above, ATP can activate P2X7 receptors to open K^+^ channels and reduce intracellular K^+^ concentration, thus promoting the activation of the NLRP3 inflammasome. When P2X7 receptors are inhibited, K^+^ outflow is blocked and a high concentration of K^+^ prevents NLRP3 inflammasome assembly and activation.

#### 3.2.2 Colchicine can damp microtubule synthesis

By immunofluorescence staining and other methods, Takuma Misawa et al. have successfully demonstrated that the assembly of not only NLRP3 and ASC but also NLRP3 inflammasomes into NLRP3 inflammasome complexes requires the transport function of microtubules.^17^ Because colchicine can inhibit the synthesis of microtubules and promote the degradation of microtubules, it can effectively inhibit the assembly of NLRP3 inflammasome complexes, and ultimately effectively inhibit the inflammatory response.

#### 3.2.3 Colchicine can effectively inhibit Caspase‐1

In the study of small intestinal injury induced by NSAID in mice, Otani K et al found that colchicine could effectively inhibit the expression of caspase‐1 and IL‐1 β, but there was no significant change in NLRP3 and pro‐IL‐1 β levels. Through the recovery experiment and NLRP3^−/−^ transgenic mice, it was further demonstrated that colchicine can inhibit inflammation by suppressing the expression of caspase‐1.^37^ In addition, Robertson S et al. collected blood from patients with acute coronary syndrome (ACS) treated with colchicine (n = 21), the non‐treated group (n = 9) and the healthy control group (n = 9). After isolation and purification of monocytes, the key marker of inflammasome and the levels of pro‐IL‐1β and pro‐caspase were analysed by enzyme‐linked immunosorbent assay (ELISA). The results showed that colchicine could decrease the levels of monocyte IL‐1 β in ACS patients by reducing the levels of pro‐caspase‐1 and caspase‐1 proteins.^38^ Caspase‐1 also plays an important role in the NLRP3 inflammasome, IL‐1β, and IL‐6 inflammatory reaction axis. It can cleave pro‐IL‐1 β and pro‐IL‐18 into their active forms. When caspase‐1 is inhibited, the level of downstream activated IL‐1 β will naturally decrease.

## 4 RESEARCH PROGRESS OF COLCHICINE IN THE TREATMENT OF ATHEROSCLEROSIS

At present, lipid‐regulating therapy is still an unshakable cornerstone in the treatment of atherosclerosis. However, inflammation runs through the onset and development of atherosclerosis. Right now, anti‐inflammatory therapy is still under exploration and has not been formally put into clinical practice. Current studies have shown that colchicine, as an anti‐inflammatory drug, is likely to become a first‐line treatment for atherosclerosis and other cardiovascular inflammatory diseases in the future.

### 4.1 Progress in Basic Research of Colchicine in the treatment of Atherosclerosis

As a classic anti‐inflammatory drug, colchicine has been widely studied in basic research, but not so much in atherosclerosis. However, with the exciting results from the large‐scale clinical trial, Colchicine Cardiovascular Outcomes Trial (COLCOT) in 2019, the status of colchicine in the study of cardiovascular inflammatory diseases has been raised to an unprecedented height.

Butt A et al. developed an abdominal aortic atherosclerosis rabbit models induced by a high cholesterol diet and balloon endothelial denudation. The 20 rabbits were divided into two groups: colchicine group and placebo control group. All rabbits were examined by MRI, F‐FDG PET/CT, optical correlation tomography (OCT) and histological assessment. The results showed that colchicine may have the effect of stabilizing atherosclerotic plaque by reducing inflammation and plaque load without changing macrophage infiltration and plaque type.^39^ The authors studied the phenotypes of atherosclerotic plaque load and plaque stability through multimodal small animal imaging methods, which would provide important reference values for follow‐up experiments.

### 4.2 Progress in Clinical Research of Colchicine in the treatment of Coronary Atherosclerotic Disease (CAD)

In the past ten years, several clinical studies were carried out to observe the therapeutic role of anti‐inflammatory drugs on cardiovascular diseases. Distinguished among them is the CANTOs trial (Canakinumab Anti‐inflammatory Thrombosis Outcome Study) in 2007. It proved that IL‐1β monoclonal antibody can effectively reduce the synthesis of liver C‐reactive protein and reduce adverse cardiovascular events.^40^, ^41^ As a classic anti‐inflammatory drug, colchicine has been used to prevent atherosclerosis in clinical trials. In 2013, Stefan M et al. conducted the Low‐dose Colchicine trail (LoDoCo trial). This was a single‐blind, randomized controlled trial that followed 532 patients with stable CAD for up to 2 years. The results showed that, compared with the control group, 0.5 mg of colchicine per day can effectively reduce the occurrence of cardiovascular events.^42^ In recent years, there have been several large‐scale clinical studies. At the end of 2019, the results of the COLCOT trial (Colchicine Cardiovascular Outcomes Trial) showed that the use of colchicine (0.5 mg per day) within 30 days after acute myocardial infarction (AMI) can reduce the risk of cardiovascular ischemic events.^1^ Compared with the LoDoCo trial, the subsequent LoDoCo2 trial in 2020 adopted a double‐blind, randomized controlled design and assigned 5522 patients with chronic coronary artery disease to either a study group taking 0.5 mg of colchicine per day or a control group taking a placebo. During the 2.4‐year follow‐up, the colchicine group showed significantly reduced spontaneous myocardial infarction, ischemic stroke, cardiovascular deaths, deficiency and PCI events caused by ischemia in patients with chronic coronary artery disease.^43^

Michelle Samuel MPH et al. obtained a total sample size of 11 594 CAD patients (colchicine n = 5774; placebo n = 5820) through databases and conducted a systematic review and meta‐analysis of randomized controlled trials, showing that in terms of secondary cardiovascular prevention, compared with the standard drug therapy alone, adding low‐dose colchicine can reduce the incidence of major cardiovascular events.^44^

However, in 2020, the COPS trial conducted a randomized, double‐blind, placebo‐controlled study on 795 ACS patients. Patients in the colchicine group took 1 mg/day of colchicine in the first month after admission to the hospital with a diagnosis of ACS. The dose of colchicine taken for the next 11 was months 0.5 mg/day. After 1 year of follow‐up study, the results of the study showed that low‐dose colchicine (0.5 mg per day) not only failed to have a significant effect on cardiovascular results, but also was associated with a higher rate of mortality.^45^ The results of this large clinical study seemed inconsistent with the positive anti‐inflammatory effects of colchicine in CVD diseases. The authors believe that colchicine can indeed effectively reduce IL‐1β, IL‐6 and CRP in the treatment of ACS patients, but long‐term use of colchicine leads to higher non‐cardiovascular mortality in ACS patients. This may be related to the dose of colchicine (0.5 mg twice per day) taken by ACS patients in the first month. Although colchicine can effectively reduce inflammation, it can easily cause adverse digestive reactions and induce infections. Because of its side effects, further clinical and basic research is needed in order to determine the safety and reliability of this medication (Table 1).

**TABLE 1:** Summary of main large‐scale clinical trials in recent years

Trail	Year	Patients	Setting	Study design	Agent dose	Main clinical results
LoDoCo^42^	2013	532	Stable CAD (n = 282), controls (n = 250)	Single‐blind RCT	Colchicine 0.5 mg/d	In patients with stable coronary disease, low dose of colchicine (0.5 mg per d) seems to effectively prevent cardiovascular events
CANTOS^40^	2017	10061	MI ≥ 30 d (n = 6717), controls (n = 3344)	Double‐blind, placebo controlled RCT	Canakinumab 50 mg, 150 mg, 300 mg/3 mo	150 mg of Canakinumab every 3 mo resulted in a significantly lower incidence of recurrent cardiovascular events than placebo, regardless of the reduction in blood lipid levels
CIRT^47^	2019	4786	Resent MI (n = 2391), controls (n = 2395)	Double‐blind, placebo controlled RCT	Methotrexate 15–20 mg/wk	Low‐dose methotrexate does not reduce IL−1β, IL−6 or C‐reactive protein levels, and does not cause fewer cardiovascular events than placebo
COLCOT^48^	2019	4745	Resent MI (n = 2366), controls (n = 2379)	Double‐blind, placebo controlled RCT	Colchicine 0.5 mg/d	0.5 mg of colchicine per day has a much lower rate of ischemic cardiovascular events than placebo
COPS^45^	2020	795	ACS and CAD (n = 396), controls (n = 399)	Double‐blind, placebo controlled RCT	Colchicine 1 mg/d for 1 mo, then 0.5 mg/day for 11 mo	The addition of colchicine to standard drug therapy has no significant effect on the cardiovascular outcome of ACS patients at 12 mo and is associated with a higher mortality rate
LoDoCo2^43^	2020	5522	Stable CAD (n = 2762), controls (n = 2760)	Double‐blind, placebo controlled RCT	Colchicine 0.5 mg/d	Patients receiving 0.5 mg of colchicine per day had a significantly lower risk of cardiovascular events than patients receiving placebo

Abbreviations: ACS, Acute coronary syndrome; CAD, coronary atherosclerotic disease; CANTOS, Canakinumab Anti‐inflammatory Thrombosis Outcome Study; CIRT, Cardiovascular Inflammation Reduction Trial; COLCOT, Colchicine Cardiovascular Outcomes Trial; COPS, The Australian COPS Trial; LoDoCo, the Low‐dose Colchicine trail; LoDoCo2, the Low‐dose Colchicine trail 2; MI, myocardial infraction; RCT, Redis Computed Tomography.

## 5 CONCLUSION AND PROSPECTS

First of all, the development of atherosclerosis is inseparable from inflammation. The inflammatory response is mainly induced by OxLDL and cholesterol crystals, which cause the assembly and activation of NLRP3 inflammasomes in macrophages, and increase the expression of downstream inflammatory factors, through the NLRP3 inflammasome, IL‐1β, IL‐6 and C‐reactive protein inflammatory response axis. As an anti‐inflammatory drug, colchicine mainly inhibits the inflammatory response in atherosclerosis in three ways, as follows: ① damping P2X7‐induced K^+^ channel opening and reduce K^+^ efflux; ② restraining microtubule synthesis and reducing the assembly of NLRP3 inflammasomes and ASC and the formation of NLRP3 inflammatory complex; ③ inhibiting caspase‐1 conversion pro‐IL‐1β into the active form of IL‐1β. Eventually, it will reduce the levels of IL‐1β, thereby inhibiting the inflammatory response in atherosclerosis.

At present, atherosclerosis is mainly treated by lipid lowering drugs such as statins are extremely widely used and have achieved very significant clinical effects. The clinical application of proprotein convertase subtilisin/kexin type 9 monoclonal antibody (PCSK9 inhibitor) has opened a new gate for lipid‐lowering therapy, but atherosclerosis is a chronic inflammatory process, and lipid‐lowering drugs cannot effectively reduce the inflammatory response. Some recent basic‐research studies have also shown that although colchicine cannot effectively restrict the development of atherosclerosis, it can effectively reduce inflammation and damp the burden of atherosclerotic plaques. In addition, colchicine also has the effect of stabilizing plaques and reducing the risk of plaque rupture.

With the continuous understanding of the mechanisms of atherosclerosis, anti‐inflammatory therapy is approaching clinical applications. From the initial IL‐1β monoclonal antibody, to methotrexate, and then to the currently studied colchicine, IL‐1β monoclonal antibodies can effectively reduce the occurrence of inflammation and adverse cardiovascular events. Although monoclonal antibodies represented by Canakinumab have been approved for clinical use in the treatment of diseases such as AOSD and SJIA, they have not yet been approved by the FDA for the treatment of cardiovascular diseases. In addition, Canakinumab is very expensive, making its widespread clinical use difficult. Colchicine, a very cheap drug, can effectively block NLRP3 inflammasomes and reduce inflammation. It has been proven in basic research to effectively reduce atherosclerotic plaque load and increase plaque stability. In multiple large‐scale clinical studies, low‐dose colchicine (0.5 mg) per day has been proven not only to reduce adverse cardiovascular events in patients with myocardial infarction, but also to effectively decrease adverse cardiovascular events in patients with chronic cardiovascular diseases.

While colchicine is anti‐inflammatory, it also comes with many side effects, such as diarrhoea and abdominal pain. When the dose of colchicine is not well controlled, it will not only cause reductions in platelets and neutrophils, and increase the risk of infection in patients,^46^ but even also induce aplastic anaemia in severe cases. These side effects and adverse reactions put certain limitations on colchicine in the anti‐inflammatory treatment of cardiovascular diseases, but we believe that as long as the dosage of colchicine is well controlled, its safety properly monitored, and a new mode of administration developed, colchicine will certainly have a very important place in the future treatment of cardiovascular inflammatory diseases.

## CONFLICT OF INTEREST

The authors declare no competing financial interest.

## AUTHOR CONTRIBUTION

**Yuyu Li:** Conceptualization (lead); Data curation (equal); Formal analysis (equal); Methodology (equal); Resources (lead); Software (lead); Supervision (equal); Validation (equal); Visualization (equal); Writing‐original draft (lead); Writing‐review & editing (equal). **Yuxin Zhang:** Investigation (equal); Methodology (equal); Validation (equal); Writing‐review & editing (equal). **Jianrong Lu:** Data curation (supporting); Formal analysis (supporting); Visualization (supporting). **Yong Yin:** Formal analysis (supporting); Supervision (supporting); Validation (supporting). **Jun Xie**: Conceptualization (equal); Project administration (equal); Supervision (equal); Validation (equal); **Biao Xu**: Conceptualization (equal); Project administration (equal); Supervision (equal); Validation (equal).

## Data Availability

Data sharing is not applicable to this article as no data sets were generated or analysed during the current study.
